# PSMD9 expression predicts radiotherapy response in breast cancer

**DOI:** 10.1186/1476-4598-13-73

**Published:** 2014-03-28

**Authors:** Fiona E Langlands, David Dodwell, Andrew M Hanby, Kieran Horgan, Rebecca A Millican-Slater, Valerie Speirs, Eldo T Verghese, Laura Smith, Thomas A Hughes

**Affiliations:** 1 Leeds Institutes of Molecular Medicine, University of Leeds, Leeds, UK; 2 St. James’s Institute of Oncology, St. James’s University Hospital, Leeds, UK; 3 Department of Histopathology, St. James’s University Hospital, Leeds, UK; 4 Department of Surgery, Leeds General Infirmary, Leeds, UK

**Keywords:** Predictive marker, Radiotherapy, Proteasome

## Abstract

**Background:**

More than 50% of cancer patients are recommended to receive radiotherapy. Recommendations are based mainly on clinical and pathological factors and not intrinsic tumour radio-sensitivity. Use of radiotherapy according to predictive markers would potentially reduce costly over-treatment, and improve the treatment risk-benefit ratio and cancer outcomes. Tumour expression of the 26S proteasome has been reported to predict radiotherapy response: low expression was associated with higher rates of local recurrence after radiotherapy, suggesting that low proteasome expression and activity was associated with radio-resistance. However, this conclusion is at odds with the emerging use of proteasome inhibitors as radio-sensitizers. Our aim was to further analyse the relevance of 26S proteasome expression, focussing specifically on the PSMD9 subunit, in the largest clinical cohort to date, and to investigate the functional role of PSMD9 in radio-sensitivity in breast cancer cell lines.

**Methods:**

We examined expression of PSMD9 using immunohistochemistry in a cohort of 157 breast cancer patients, including 32 cases (20.4%) that subsequently developed local recurrences. The value of expression as a prognostic or radiotherapy predictive marker was tested using Kaplan-Meier and Cox regression analyses. PSMD9 function was examined in breast cancer cell lines MCF7 and MDA-MB-231 using siRNA knock-downs and colony forming assays after irradiation.

**Results:**

Low tumour PSMD9 expression was significantly associated with a reduced incidence of local recurrence in patients receiving adjuvant radiotherapy (univariate log rank p = 0.02; multivariate regression p = 0.009), but not in those treated without radiotherapy, suggesting that low PSMD9 expression was associated with relative tumour radio-sensitivity. In support of this, reduction of PSMD9 expression using siRNA in breast cancer cell lines *in vitro* sensitized cells to radiotherapy.

**Conclusions:**

We conclude that PSMD9 expression may predict radiotherapy benefit, with low expression indicative of relative radio-sensitivity, the opposite of previous reports relating to 26S proteasome expression. Our conclusion is compatible with use of proteasome inhibitors as radio-sensitizers, and highlights PSMD9 as a potential target for radio-sensitizing drugs.

## Background

Radiotherapy (RT) is a critical component of loco-regional cancer management and is used in over 50% of cancer patients [1]. RT treatment decisions are based on clinical factors, morphology-based pathological indicators, the extent of surgery, and/or clinician and patient choice rather than tumour molecular profiles predictive of recurrence and likely RT sensitivity. As a result, some patients are treated with RT although their tumours are relatively resistant, and these patients are unlikely to derive therapeutic benefit. This represents over-treatment in terms of resources and contributes to treatment-induced morbidity [2]. Biomarkers indicative of likely RT response would allow therapy to be assigned more effectively [3] yet development of such markers has progressed little [4]. Several potential markers can be identified from the literature: for example, in breast cancers expression levels of HJURP mRNA can predict patient survival after RT [5] and high cytoplasmic expression of peroxiredoxin-I correlated with increased local recurrences after RT [6]. However, in most cases predictive and prognostic information from potential markers has not been separated and markers have typically not been validated in further studies. Consequently, predictive markers for RT are not close to entering clinical practice [3].

One potential predictive marker of interest is the 26S proteasome; this has been associated with patient outcome after RT in two independent studies and, in addition, is itself a target for cancer therapeutics. The 26S proteasome is a 2.6 MDa complex of at least 47 polypeptide subunits [7], and the complex is responsible for the degradation up to 80% of cellular proteins [8]. Work with respect to RT response has focused on expression of the p32 subunit of the 20S core particle of the proteasome. Unfortunately, the gene encoding this subunit and therefore its exact molecular role remain unclear. Further confusion concerning the antibody used and its target has derived from the target being referred to as “the 26S proteasome”, as opposed to as the individual specific subunit, or in some sources as a different proteasomal subunit, p27. Low p32 expression in tumours was significantly associated with an increase in local recurrence in laryngeal cancer patients who were treated with single modality RT with curative intent [9]. Similarly, low expression of p32 was significantly associated with increased local recurrences in a small cohort of breast cancer patients who were treated with adjuvant RT [10]. Thus, it was inferred that low 26S proteasome function was associated with relative resistance to RT. However, this correlation is potentially at odds with the apparent function of proteasome inhibitors as cancer therapeutics. Other studies have shown that inhibition of 26S proteasome function can render tumour cells particularly sensitive to DNA damaging agents including ionising radiation [11,12], and can reduce tumour cell proliferation and induce apoptosis [13]. Accordingly, the proteasome inhibitor Bortezomib (Velcade; Millenium Pharmaceuticals) is now used in the treatment of some haematological malignancies [14,15] and is undergoing trials for use in other cancers, including solid tumours [16]. The conflict is that intrinsically low 26S proteasome levels (or at least of the p32 subunit) appear to correlate with RT resistance and therefore local recurrence [9,10], while reduction of 26S proteasome function sensitizes tumour cells to RT and reduces cancer cell viability. Our aim in this study was to analyse further the value of the 26S proteasome as a predictive marker for RT and a prognostic marker for local recurrence in breast cancer using a cohort that is substantially larger than any previous study. We focused on a different subunit, PSMD9, which is an associated component of the 19S regulatory particle of the 26S proteasome [7]. This was because we were able to validate the specificity of an antibody against this subunit, and we were able to use RNA interference to manipulate expression *in vitro* since the encoding gene is known. We also aimed to investigate *in vitro* whether the PSMD9 proteasomal subunit may represent a more specific target for radio-sensitizing therapies.

## Results

### PSMD9 expression predicts response to RT in breast cancers

**Figure 1:**
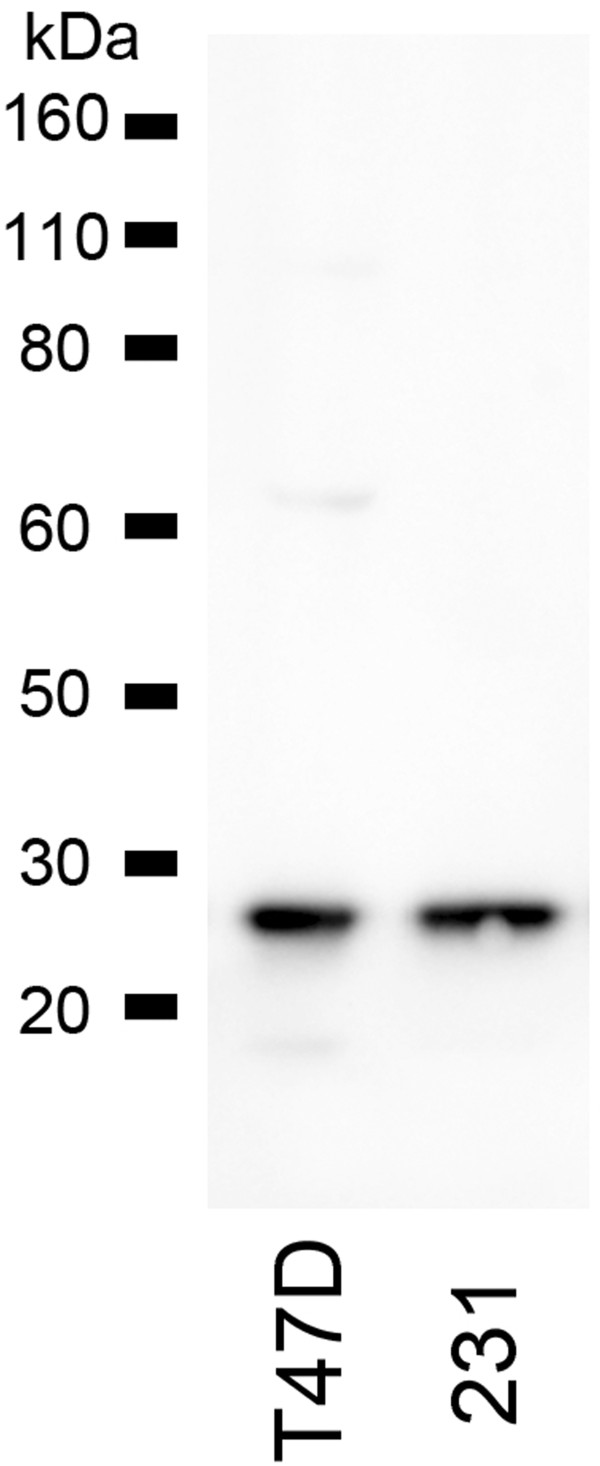
The anti-PSMD9 antibody used in this study recognises only one protein, which is of ~25 kDa - the predicted size for PSMD9, in breast cancer cell lines.

First, we selected and validated an antibody with the appropriate specificity for a subunit of the 26S proteasome. We were unable to demonstrate the specificity for p32 of the clone used in the previously published work [9,10], therefore we selected a different antibody against the PSMD9 subunit. We performed Western blot analyses to confirm that our antibody recognised a protein of the appropriate size for PSMD9 (~25 kDa) in breast cancer cell lines (Figure 1). Critically, the antibody recognised only a single protein species of the correct size demonstrating that it did not cross-react with other proteins in breast epithelial cells and was therefore potentially suitable for use in immunohistochemistry of tissues.

**Figure 2:**
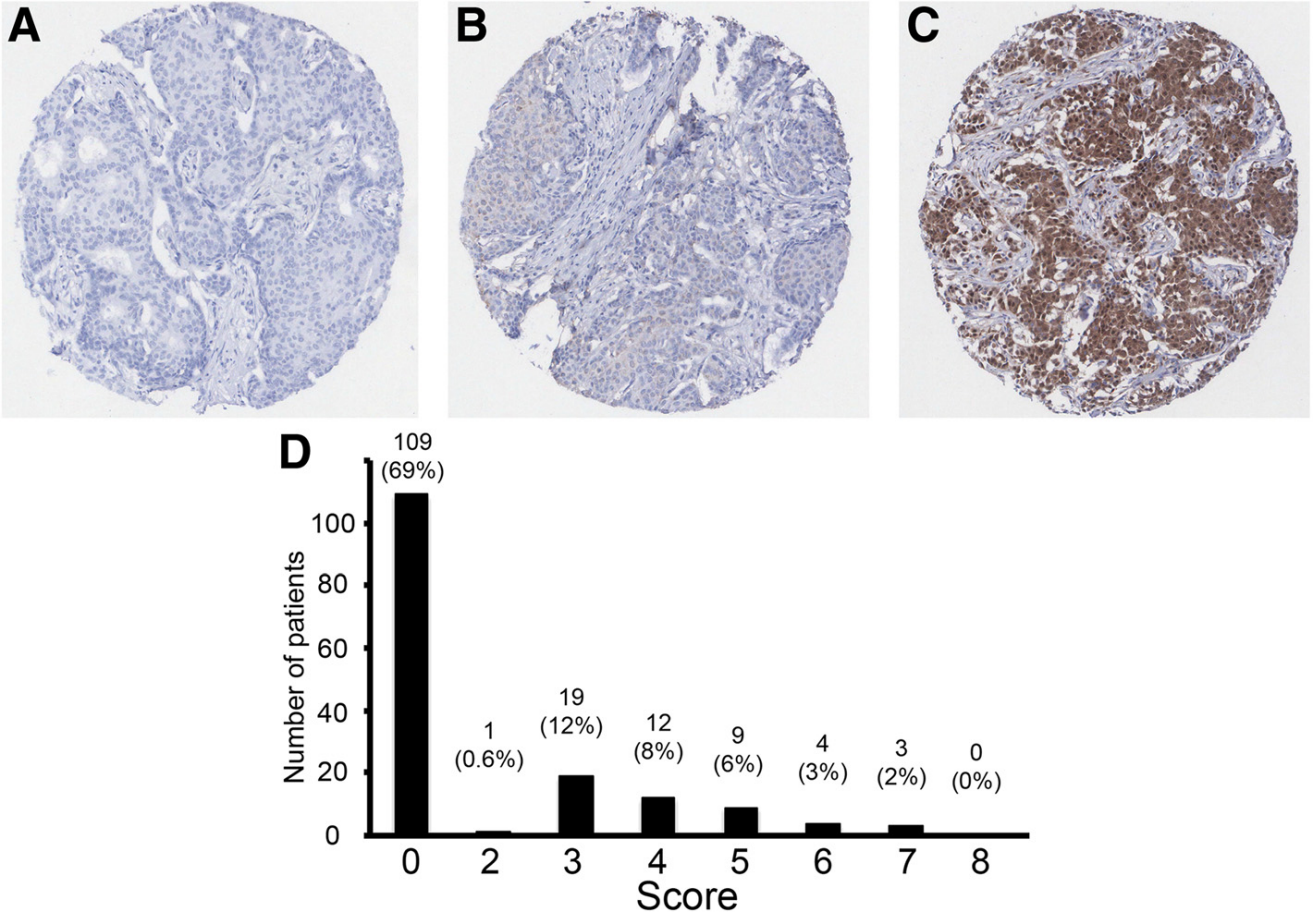
**PSMD9 is variably expressed in breast cancers. A**-**C** Representative staining patterns in individual tissue microarray cores. Cores showing negative staining **(A)**, and staining scored 3 **(B)** or 7 **(C)** are shown. **D** A histogram showing the distribution of immunohistochemistry scores. Scores (x-axis) and numbers of cases assigned to each score (y-axis) are shown. Numbers and percentages of the cohort are given above each bar.

Next, our aim was to examine whether PSMD9 expression in tumours was associated with response to RT. We have taken local recurrences (LRs) after resection surgery and adjuvant RT to be indicative of poor responses to RT. LRs of breast cancers are relatively uncommon, occurring in less than 7% of patients after 5 years [2], therefore it was not possible to use a sequential cohort of breast cancers to test the relationship between PSMD9 expression and LR without the cohort being prohibitively large. We assembled a cohort of primary breast tumours that was selected to contain a higher proportion of tumours that recurred locally (20.4%). Expression of PSMD9 was examined using immunohistochemistry in these 157 breast tumours, taking into account the proportion of tumour cells staining positively, and their intensity using the Allred system [17]. Representative stained tissue samples are shown in Figure 2A-C. PSMD9 staining was absent in most cases (69.4%), while positive staining varied from weak to strongly positive in differing proportions of cells (Figure 2D). Clinico-pathological data for the patients and tumours are described in Table 1. PSMD9 expression did not correlate significantly with patient age or with tumour characteristics (grade, size, receptor status, lymph node status; Additional file 1: Table S1).

**Table 1:** Clinico-pathological features of the breast cancer cohort (n = 157)

**Characteristic**	**Category**	**Number of cases (%)**
Age (years)	Median: 59	
	Range: 31-93	
Surgery	Wide local excision	89 (56.7)
	Mastectomy	68 (43.3)
Grade	1	29 (18.5)
	2	73 (46.5)
	3	55 (35.0)
Size (cm)	<2	73 (46.5)
	2-5	69 (43.9)
	>5	15 (9.6)
Hormone receptors	ER +	111 (70.7)
	ER -	46 (29.2)
	HER2 +	4 (2.5)
	HER2 -	18 (11.5)
	HER2 unknown	135 (86.0)
LN status (number positive nodes)	0	86 (54.8)
	1-3	40 (25.5)
	4+	26 (16.6)
	Unknown	5 (3.2)
Adjuvant therapy	Chemotherapy	40 (25.5)
	No chemotheray	117 (74.5)
	Endocrine	115 (73.2)
	No endocrine	29 (18.5)
	Endocrine unknown	13 (8.3)
	RT	110 (70.1)
	No RT	47 (29.9)
Local recurrence	Yes	32 (20.4)
	No	125 (79.6)
Systemic recurrence	Yes	49 (31.2)
	No	108 (68.8)
Follow-up (months)	Median: 96	
	Range: 9-220	

**Figure 3:**
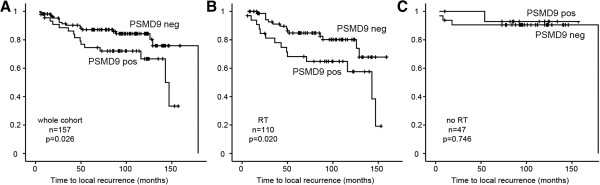
**Positive expression of PSMD9 is significantly associated with higher rates of local recurrences after RT in breast cancer.** Kaplan–Meier analyses for local recurrence in patient groups with tumours showing positive (any appreciable; “pos”) or negative (no appreciable; “neg”) staining for PSMD9. **A** A mixed group of 157 patients. **B** Patients treated with RT. **C** Patients not treated with RT.

PSMD9 expression was dichotomised in order to allow Kaplan-Meier analyses of the influence of PSMD9 on LR. Receiver operator curve analysis was performed in order to select a cut off objectively that gives the best balance between sensitivity and specificity for the end point of LR, and therefore allows the strongest use of these scoring data (Additional file 2: Figure S1). A cut off of 1 was selected, thereby defining tumours with no staining for PSMD9 as negative, and those with any staining as positive. Kaplan-Meier analyses were performed to study influences of PSMD9 expression on LR in the entire cohort (Figure 3A). Positive expression of PSMD9 was significantly associated with an increased LR rate (p = 0.03), suggesting that PSMD9 provides prognostic information. Moreover, when the cancer cohort was divided into patients treated with RT (n = 110) and patients treated without RT (n = 47) insights were gained into the predictive value of PSMD9 for RT (Figure 3B and C). It should be noted that type of surgical operation (wide local excision vs mastectomy) and lymph node status were the only significant clinico-pathological differences between the groups treated with or without RT (Additional file 3: Table S2); tumour size, grade or receptor expression did not differ significantly. Lymph node positivity and surgery type are used to select patients for RT, therefore these differences were expected. Positive expression of PSMD9 was significantly associated with LR specifically in patients who received RT (Figure 3B, p = 0.02), but not in those who did not receive RT (Figure 3C, p = 0.75).

Multivariate regression analysis was performed to assess whether the predictive value of PSMD9 was independent of standard prognostic characteristics in the cohort treated with RT. Variables included were tumour size and grade, lymphovascular invasion, lymph node status, and PSMD9 expression. Only PSMD9 expression was significantly associated with LR in this analysis, with positive expression increasing LR risk by a hazard ratio of 2.9 (95% confidence intervals 1.3-6.4; p = 0.009), indicating that PSMD9 gave predictive insights that were unrelated to standard prognostic factors.

### Knock-down of PSMD9 sensitises breast cancer cells to RT

**Figure 4:**
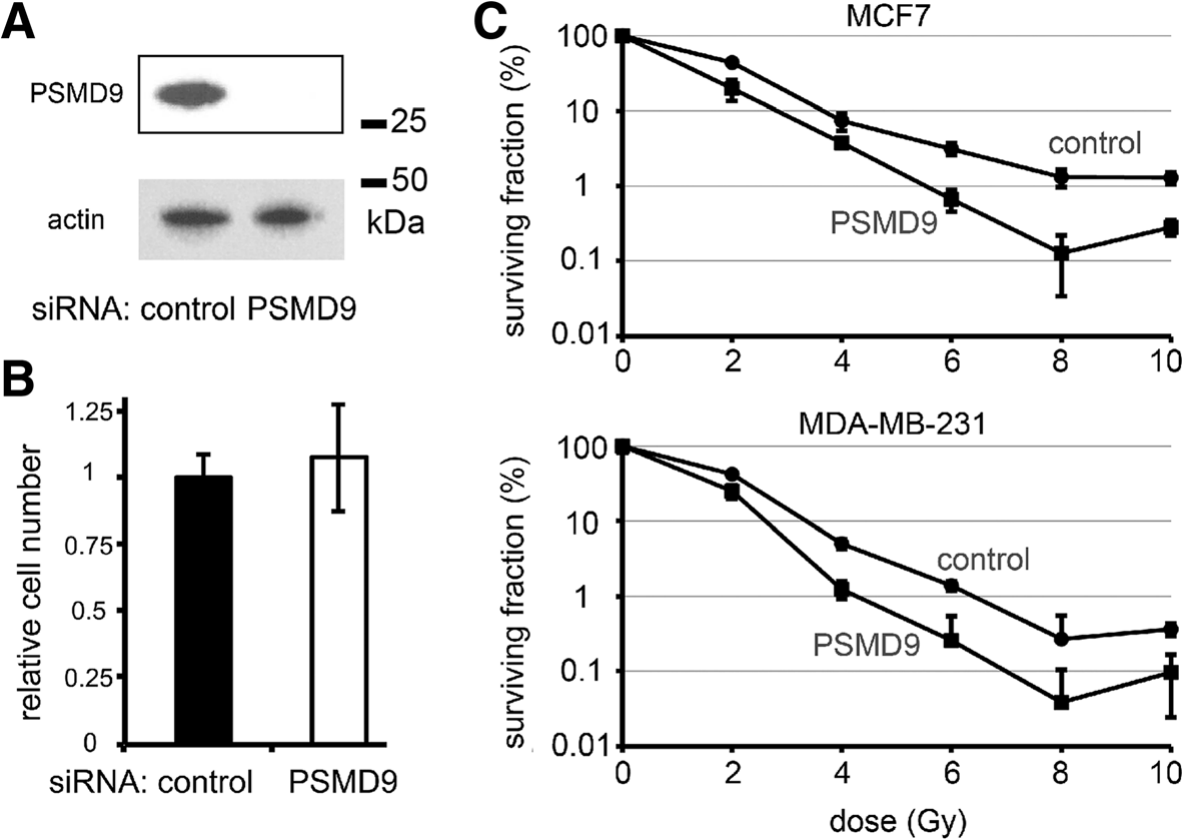
**PSMD9 expression is functionally associated with RT response in breast cancer cells.** Breast cancer cell lines were transiently transfected with siRNAs targeting PSMD9 or with non-targeting control. **A** PSMD9 was effectively silenced using siRNA, as demonstrated using Western blotting of MCF7 transfected lysates. **B** MCF7 cell growth after 48 h was unaffected by PSMD9 knock-down, as demonstrated using MTT survival/proliferation assays. **C** PSMD9 knock-down enhanced the efficacy of RT in breast cancer cell lines, as demonstrated using colony forming assays. Data points represent means of triplicates (+/- standard deviations).

In order to determine whether PSMD9 expression was functionally associated with response to RT, we manipulated PSMD expression using siRNA in breast cancer cell lines and assessed sensitivity to RT *in vitro*. First, MCF7 cells were transiently transfected with siRNAs targeting PSMD9 or with a non-targeting control, and PSMD9 expression was examined using Western blotting (Figure 4A). Expression of PSMD9 was dramatically reduced by the targeted siRNAs. PSMD9 knock-down had no significant influence on cell survival/growth 48 h after transfection (Figure 4B) suggesting PSMD9 expression has little influence on the short-term survival of the bulk population of cells. Next, we performed colony-forming assays with breast cancer cell lines representative of both luminal A (MCF7) and the basal (MDA-MB-231) subtypes after transfection with PSMD9-targeting siRNA, or control, and after different doses of radiation from 0 to 10 Gy (Figure 4C). Cells surviving irradiation and maintaining sustained proliferative potential were quantified by counting individual colonies. Knock-down of PSMD9 significantly sensitized both cell lines to all doses of radiation (p < 0.05; except MCF7 cells at 4 Gy and MDA-MB-231 at 8 Gy, which did not reach statistical significance). The proportion of cells surviving RT was reduced by up to 10-fold after PSMD9 knock-down.

## Conclusions

We show that low expression of the 26S proteasome subunit PSMD9 was significantly associated with reduced incidence of LR in breast tumours after adjuvant RT. Our results for PSMD9 are, in essence, opposite to those of previous studies on the prognostic/predictive value of the expression of the proteasomal subunit p32 in cancers, in which low p32 expression was found to associate with increased LR rates after RT [9,10]. Two factors are worthy of discussion with respect to this apparent conflict. Firstly, and most obviously, the reports focus on different subunits of the proteasome and it is plausible that the two subunits have opposite correlations with RT response. It remains unclear whether expression of either is representative of proteasomal activity, as would be relevant when considering therapies with proteasome inhibitors. Secondly, we have used a cohort of cancer cases (n = 157) that is substantially larger than those in the previous studies (n = 47 [9] and n = 28 [10]) therefore our result may be more robust. A further key point related to this is that we are the first to attempt to separate the predictive and prognostic aspects of expression of a proteasomal subunit (Figure 3). The previous studies used cohorts where all patients received RT [9,10] therefore it was not possible to determine whether expression correlated with outcome irrespective of treatment (prognostic) or with RT response (predictive). In breast cancers, we found PSMD9 expression to correlate with outcome only in patients treated with RT (Figure 3B), indicating that PSMD9 represents a RT predictive marker rather than a simple prognostic marker for LR. While statistically significant, this result is potentially flawed by the low LR rate in patients who did not receive RT (Figure 3C). This flaw is unfortunately experimentally intractable for breast cancer since only patients at a low risk of LR are spared RT (for example, those with negative lymph nodes after mastectomy) therefore LRs are inevitably very uncommon in the no RT group. A further difficulty that follows from this is that the groups that receive and do not receive RT differ in terms of their lymph node status, although not in other pathological parameters (Additional file 3: Table S2).

The need for RT predictive markers has long been highlighted [4]. It is well established that breast cancer subtypes (as defined by estrogen receptor, progesterone receptor, and her-2 expression) correlate with LR rates [18,19], with the highest rates in the estrogen receptor negative subtypes. There is evidence that these differences in LR relate to differential responses to RT [20], although this is not well accepted enough to be taken into account when stratifying patients for RT. We do not find PSMD9 expression to correlate with estrogen receptor expression (Additional file 1: Table S1), therefore we do not believe it to be involved with the differential RT response between these groups; we can not comment on the relationships between her-2, PSMD9 and response, as her-2 data are lacking for our cohort, which largely pre-dates the start of routine her-2 testing in the UK. It is notable that development of specific molecular predictive markers for RT has lagged behind that of predictive markers for modern, targeted, systemic therapies, for which the therapeutic target itself, or molecules associated with it, have often proved good candidate markers. Recently the search for RT predictive markers has followed an analogous course, focussing mainly on proteins involved with DNA metabolism and repair, since DNA is the target of RT. Some potential markers have been identified [5,21,22] but none have yet entered clinical practice. In addition, The mechanism by which PSMD9, or the 26S proteasome generally, is associated with RT response remains unclear although emerging evidence suggests that the proteasome may be an upstream regulator of some of key DNA repair pathways [23], with low proteasome activity reducing DNA repair capacity and causing relative RT sensitivity. In particular, efficient proteasome function is potentially required for homologous recombination, a pathway for repairing RT-induced DNA damage, and for post-replication repair [23], providing a mechanistic framework to support the emerging use of proteasome inhibitors as radio-sensitizers [24,25]. The 26S proteasome inhibitor, Bortezomib (Velcade; Millenium Pharmaceuticals), has shown a positive clinical benefit for inducing radio-sensitization in some cancers, yet the majority of success remains in haematological malignancies and its influence on solid tumours has been less encouraging [26]. Clinical use of Bortezomib continues to be hampered by dose-limiting toxicities, drug-resistance and interference by some natural compounds. Our cell culture work supports the proposition that the PSMD9 subunit itself represents a specific and novel target for radio-sensitizing therapies, since transiently reduced PSMD9 expression at the time of exposure to RT was associated with reduced cell survival (Figure 4).

In conclusion, we show that PSMD9 expression may be a predictive marker for RT in breast cancer. This observation warrants further prospective evaluation, ideally in the context of prospective randomized studies of RT, to determine whether PSMD9 expression has clinical utility in targeting RT to those patients most likely to benefit, or in selecting patients with relatively RT resistant tumours for more intensive therapy. We also demonstrate that PSMD9 may itself present a therapeutic target for a further generation of subunit-specific proteasome inhibitors.

## Methods

### Ethical issues and study populations

Ethical approval was obtained from Leeds (Central) Research Ethics Committee (reference 08/H1313/49). The cohort comprised patients treated at the Leeds Teaching Hospital NHS Trust for operable primary breast cancer between 1993 and 2007 and either having suffered local recurrences (LRs) (n = 32), or having not had LRs (n = 125; minimum follow up for remaining LR free: 52 months), and for whom archival primary resection blocks and clinical follow up were available. Extensive clinical follow up data, including time from initial diagnosis to either LR or last follow up, were collected from patient notes and from Trust computer databases. LRs were defined as such if they occurred in the same breast quadrant as the index lesion for conserved breasts or in association with the mastectomy scar, and with the same histology (type, grade) and hormone receptor status. Patients were treated with surgery (wide local excision/mastectomy) with or without adjuvant RT. RT, when given, was 40 Gy in 15 fractions over 3 weeks. A variety of other standard adjuvant therapies were given (Table 1); these were typically oral anti-hormonal agents and/or chemotherapy.

### Tissue culture, siRNA transfection and MTT assays

Cell lines were obtained from the European Collection of Animal Cell Cultures and were cultured as described previously [27,28]. Bimonthly Mycoplasma checks (MycoAlert Mycoplasma detection assay, Lonza [Basal, Switzerland]) were consistently negative and short tandem repeat profiles confirmed cell identity. SiRNAs were purchased from Dharmacon (Waltham, USA) or Ambion (Paisley, UK). The siRNA against PSMD9 is pool of three independent sequences all directed against PSMD9. Cells were reverse transfected with 50 nM targeting or non-targeting siRNAs using Lipofectamine RNAiMax (Invitrogen [Paisley, UK]). MTT (Dimethylthiazol diphenyltetrazolium bromide) assays were performed as previously described [29].

### Western blot analyses

RIPA lysates (50 mM Tris HCl pH7.4, 150 mM NaCl, NP40 1%, Complete inhibitors [Roche, Basel, Switzerland]) were prepared from cells and were quantified in triplicate with RCDC protein assay (BioRad, Hercules, USA). Western blot analyses were performed as previously [30] using 25 μg protein per lane on SDS 4–15% polyacrylamide gels (Invitrogen, Paisley, UK) and Hybond-ECL membrane (Amersham Biosciences, Buckinghamshire, UK). Antibodies and conditions used were mouse anti-26S proteasome (1:400, 4°C, 16h [Abcam Ab58115, Cambridge, UK]), mouse anti-actin (1:5000, 20°C, 1 h [Sigma A1978, Poole, UK]) and goat anti-mouse HRP-conjugated secondary (1:1000, 20°C, 1 h [Santa-Cruz Biotech sc-2005, Santa Cruz, USA]). Proteins were visualised with Super Signal West Femto Substrate (Thermo Scientific, USA) and a ChemiDoc Gel Documentation System using Quantity One software (version 4.6.1) (Bio-Rad, Hercules, CA, UK).

### Immunohistochemistry, pathology scoring, and statistics

Tissue microarrays (TMAs) were constructed using 0.6mm cores selected from the most representative tumour areas (determined by analysis of H&E stained sections by breast pathologists ETV, RAM-S and AMH) of formalin-fixed paraffin-embedded (FFPE) archival resection tissue blocks of primary tumours. 3 separate cores were taken from different areas of each tumour. Liver and kidney tissues were also placed within TMAs to allow orientation, to act as internal controls, and to assess equal staining across slides. TMAs were sectioned onto SuperFrost Plus slides (Menzel-Glaser, Braunschweig, Germany). Sections were dewaxed with xylene and rehydrated through graded ethanol before blocking of endogenous peroxidase activity in 3% H_2_O_2_ (10 min). Epitopes were retrieved by heating in a pressure-cooker in 1% vector antigen unmasking solution (2 min) and non-specific binding blocked using 10% Casein solution (20 min). Slides were incubated with mouse anti-26S proteasome (Ab58115, Abcam, Cambridge, UK) at a dilution of 1:300 for 16 h at 4°C. Staining was visualised using Envision kits (Dako, Gostrup, Denmark). Slides were washed in tris-buffered saline and stained in copper sulphate, Harris’s haematoxylin and finally in Scotts substitute for 1 min before dehydration. Slides were mounted in DPX (Fluka, UK). Stained sections were digitally scanned using Scanscope XT (Aperio) at 40x magnification and were observed using ImageScope (Aperio). Staining of tissues was scored for immunoreactivity by two independent observers (FEL and RAM-S) taking into account intensity and percentage of positively stained tumour cells using the Allred system [17]. Staining intensity scores (0, no staining; 1, weak; 2, moderate; 3, strong) were added to percentage positivity scores (0, 0%; 1, <1%; 2, 1-5%; 3, 6–25%; 4, 26–75%; 5 > 75%) giving totals of 0 or 2–8. When scores given by individuals differed substantially consensus scores were determined by consultation with a further independent consultant breast pathologist (AMH). Statistical analyses were performed using SPSS v16.0 with tests (two tailed) as described in the text. P values of less than 0.05 were considered to indicate significance.

### Colony-forming assays

These were performed essentially as described previously [9]. Cell lines were transfected in T25 cm^2^ tissue culture flasks and cultured for 48 h as normal. Irradiation was then performed using a 320 System Irradiator (320 kV x-ray source; NDT Equipment Suppliers, UK). Cells were irradiated with single fractions of 0, 2, 4, 6, 8 and 10 Gy and cultured as normal for a further 4 h. Each flask of cells was then seeded into triplicate 10 cm tissue culture plates. Cells were seeded at different densities according to cell type and radiation exposure in order to achieve an assessable number of colonies. The cells were then cultured undisturbed for 14 days. Cells were fixed and stained in 5 mg/ml Crystal violet, 50% Methanol, 20% Ethanol (20°C, 20 s) before being rinsed in water twice. Colonies were then counted using Quantity One 1-D analysis Software (BioRad) version 4.6. Calculation of survival fractions (SF) was performed using the equation: SF = colonies counted/cells plated × (PE/100), where PE is a measure of individual plating efficiency.

## Competing interests

The authors declare that they have no competing interests.

## Authors’ contributions

FEL and LS performed the wet-lab experiments. FEL, AMH, RAM-S and ETV were involved were the histopathology scoring. FEL, DD, AMH and KH collected archival samples and/or clinical data. FEL and ETV performed the statistical analyses. DD, AMH, KH, VS, LS and TAH conceived of and the directed the project. All authors contributed to writing the manuscript. All authors read and approved the final manuscript.

## Authors’ information

The authors comprise specialists in all components of the multi-disciplinary team for clinical breast cancer treatment, including surgeons (FEL and KH), an oncologist (DD) and histopathologists (AMH, RAM-S and ETV). LS, VS and TAH are molecular cancer biologists specialising in the study of breast cancer.

## Supplementary Material

Additional file 1: Table S1 PSMD9 expression does not correlate with clinico-pathological features of the breast cancer cohort (n = 157). Click here for file

Additional file 2: Figure S1 Receiver Operator Curve analysis was used to select a cut-off to dichotomise PSMD9 expression. Click here for file

Additional file 3: Table S2 Lymph node (LN) status and surgery type, but not other clinico-pathological features, differ significantly between breast cancer patients treated with (+) and without (-) radiotherapy. Click here for file
