# Supplementary material for: PSMD9 expression predicts radiotherapy response in breast cancer
Source: Mol Cancer. 2014 Mar 28;13:73. doi: 10.1186/1476-4598-13-73 (PMC4230020; doi:10.1186/1476-4598-13-73)
Supplement: Additional file 1: Table S1 — PSMD9 expression does not correlate with clinico-pathological features of the breast cancer cohort (n = 157). [file 1476-4598-13-73-S1.doc]

| **Patient/tumour characteristic** | **Spearman’s rho coefficient**  **(p value)** |
| --- | --- |
| Age at diagnosis | 0.090  0.280 |
| Grade | -0.107  0.196 |
| Size | 0.159  0.055 |
| Lympho-vascular invasion | 0.008  0.923 |
| Estrogen receptor positivity | 0.009  0.916 |
| Progesterone receptor positivity | -0.132  0.336 |
| Nottingham Prognostic Index | 0.009  0.909 |
| Lymph node status | 0.115  0.173 |
| Number nodes positive | 0.115  0.173 |

**Table S1** PSMD9 expression does not correlate with clinico-pathological features of the breast cancer cohort (n=157).
