# Supplementary figures and images for: PSMD9 expression predicts radiotherapy response in breast cancer
Source: Mol Cancer. 2014 Mar 28;13:73. doi: 10.1186/1476-4598-13-73 (PMC4230020; doi:10.1186/1476-4598-13-73)

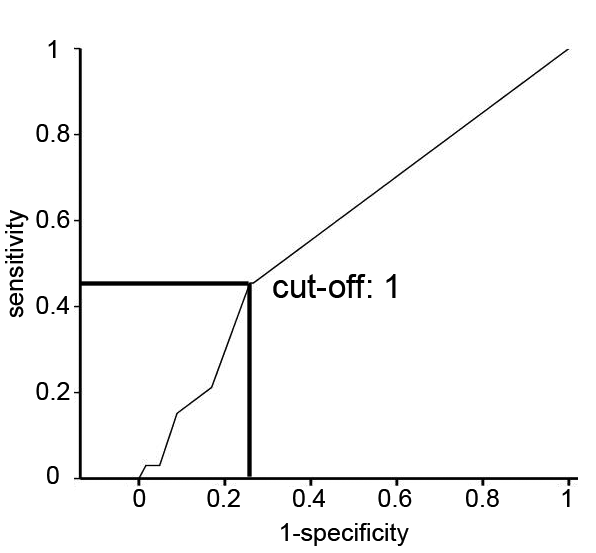

Supplement: Additional file 2: Figure S1 — Receiver Operator Curve analysis was used to select a cut-off to dichotomise PSMD9 expression. [file 1476-4598-13-73-S2.tiff]
