# Supplementary material for: PSMD9 expression predicts radiotherapy response in breast cancer
Source: Mol Cancer. 2014 Mar 28;13:73. doi: 10.1186/1476-4598-13-73 (PMC4230020; doi:10.1186/1476-4598-13-73)
Supplement: Additional file 3: Table S2 — Lymph node (LN) status and surgery type, but not other clinico-pathological features, differ significantly between breast cancer patients treated with (+) and without (-) radiotherapy. [file 1476-4598-13-73-S3.doc]

| **Characteristic** | **Category** | **Cases +RT n=110 (%)** | **Cases -RT n=47 (%)** | **Mann-Whitney** |
| --- | --- | --- | --- | --- |
| Age (years) |  | median:  58 | median:  59 | 0.0536 |
| Surgery | wide local excision  mastectomy | 78 (70.3)  33 (29.7) | 12 (25.5)  35 (74.5) | <0.0001 |
| Grade | 1  2  3 | 18 (16.4)  48 (43.6)  44 (40.0) | 10 (21.3)  25 (53.2)  12 (25.5) | 0.106 |
| Size (cm) | <2  2-5  >5 | 55 (50.0)  44 (40)  11 (10) | 23 (48.9)  22 (46.8)  2 (4.3) | 0.2885 |
| Hormone receptors | ER +  ER -  HER2 +  HER2 -  HER2 unknown | 72 (65.5)  38 (34.5)  4 (3.6)  16 (14.5)  90 (81.8) | 38 (80.9)  9 (19.1)  0 (0)  2 (4.3)  45 (95.7) | 0.06  0.55 |
| LN status (number positive nodes) | 0  1-3  4+  Unknown | 56 (50.9)  24 (21.8)  26 (23.6)  4 (3.6) | 34 (72.3)  13 (27.7)  0 (0)  0 (0) | 0.0033 |

**Table S2** Lymph node (LN) status and surgery type, but not other clinico-pathological features, differ significantly between breast cancer patients treated with (+) and without (-) radiotherapy.

**Table 1** Clinico-pathological features of the breast cancer cohort (n=157).
